# Inflammation Modifies miR-21 Expression Within Neuronal Extracellular Vesicles to Regulate Remyelination Following Spinal Cord Injury

**DOI:** 10.1007/s12015-023-10560-y

**Published:** 2023-05-31

**Authors:** Tianyu Han, Peiwen Song, Zuomeng Wu, Yunlei Liu, Wang Ying, Cailiang Shen

**Affiliations:** 1 Department of Orthopedics (Spinal Surgery), The First Affiliated Hospital of Anhui Medical University, 218 Jixi Road, Shushan District, Hefei City, Anhui Province China; 2 Department of clinical laboratory, People’s Hospital of Fuyang, Fuyang, China; 3 Department of Medical Imaging, The First Affiliated Hospital of Anhui Medical University, Hefei, China

**Keywords:** Spinal cord injury, Neural stem cells, Neuron, Extracellular vesicles

## Abstract

Cell‒cell communication following spinal cord injury (SCI) plays a key role in remyelination and neurological recovery. Although communication between neuron-neural stem cells (NSCs) affects remyelination, its precise mechanism remains unclear. The present study investigated the biological effects of extracellular vesicles (EVs) derived from neurons on the differentiation of NSCs and the remyelination of axons in a rat model for SCI. We found that that EVs derived from neurons promoted the differentiation of NSCs into oligodendrocytes and the remyelination of axons in SCI rats. However, neuron-derived EVs lost their biological effects after inflammatory stimulation of these neurons from which they originate. Further analysis demonstrated that the inflammatory stimulation on neurons upregulated miR-21 within EVs, which targeted SMAD 7 and upregulated the TGF-β/SMAD2 signaling pathway, resulting in an excess of astrocytic scar boundaries and in remyelination failure. Moreover, these effects could be abolished by miR-21 inhibitors/antagomirs. Considered together, these results indicate that inflammatory stimulation of neurons prevents remyelination following SCI via the upregulation of miR-21 expression within neuron-derived EVs, and this takes place through SMAD 7-mediated activation of the TGF-β/SMAD2 signaling pathway.

**Supplementary Information:**

The online version contains supplementary material available at 10.1007/s12015-023-10560-y.

**Figure:**
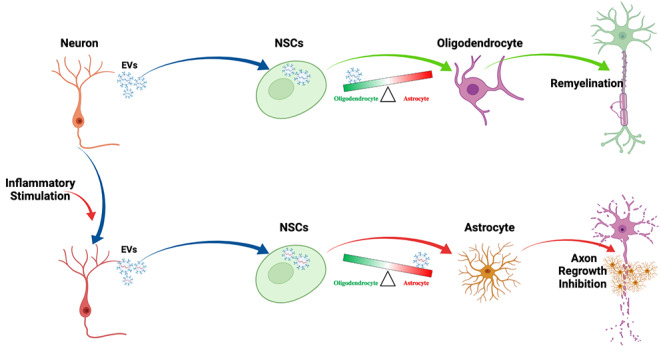
Graphical Astract

## Introduction

Spinal cord injury (SCI) causes irreversible axonal and neuronal death as well as axonal demyelination, resulting in permanent neurological dysfunction [1, 2]. Primary injury to the spinal cord directly causes the death or apoptosis of neurons or axons [3, 4], whereas the posttraumatic inflammatory process following primary injury plays a critical role in secondary damage after SCI, resulting in demyelination, axonal dieback, and further neurological function loss [3–6].

In adult mammals with a fully–developed central nervous system (CNS), the remyelination capacity and regenerative capacity of neurons and axons remain at a relatively low level, limiting spontaneous neural circuit reorganization following SCI. However, the discovery of endogenous neural stem cells (NSCs) has raised the promising possibility of axonal remyelination and regeneration of neurons and axons following SCI [7]. NSCs have been reported to exist in the CNS of all adult mammals, and they can be activated and migrate to lesion sites following SCI [7]. NSCs, like other stem cell types, have the abilities of self-renewal and multipotency, which means they can differentiate into mature nerve cells. These endogenous NSCs were long thought to be able to either contribute to the remyelination of axons or replace the damaged axons and neurons, compensating for the injury-induced cell loss and allowing for the reorganization of neural circuits and neurological recovery after SCI [8, 9]. However, further study found that most of these endogenous NSCs differentiated into astrocytes forming glial scars rather than into neurons or oligodendrocytes [10–12]. The excess formation of these astrocytic scars might prevent the remyelination or regrowth of axons following SCI [4]. Therefore, understanding and being able to manipulate the mechanism of the differentiation of NSCs following SCI is critical for the regeneration of axons and their remyelination.

Recent studies have pointed out that multicellular extracellular vesicles (EVs)-induced crosstalk is closely associated with the progression of pathological features following neurotrauma. EVs are membrane-delimited particles that are secreted by nearly all cell types and participate in multiple pathological processes by delivering bioactive cargoes (including proteins, microRNAs, and lipids) that regulate the biological functions of adjacent recipient target cells [13–21]. Although the understanding of this cell‒cell communication following neurotrauma is limited, emerging evidence reports that neuron-induced communication with target cells plays a significant role in the inflammatory process, axonal regeneration/remyelination, and glial scar formation [19, 22–25].

A variety of miRNAs are present within cell-derived EVs [26, 27], and a recent study found that the miRNAs composition of the EVs could be altered in response to external stimuli [19, 28, 29]. Among these miRNAs, miRNA-21 is not only considered to be a key switch in the inflammatory response, but also to be associated with glial scar formation and myelination [30–34]. In a study of peripheral axon injury, miRNA-21 in EVs derived from neurons was increased in response to damage caused by capsaicin stimulation. Furthermore, these neuron-derived EVs could be phagocytized by adjacent macrophages, leading to an increase in the proinflammatory phenotype [29]. Another study showed that the expression of miRNA-21 in astrocytes was upregulated around the lesions following SCI, enhancing their hypertrophic phenotype of astrocytes. In contrast, inhibition of miRNA-21expression reduced the size of astrocytes and was accompanied by increased axonal density around the injured lesion site, indicating that miRNA-21 plays a novel role in the regulation of astrocytic scar formation and axonal regeneration following SCI [30]. Therefore, we questioned whether inflammation could stimulate neurons to increase the levels of miRNA-21 within EVs and whether these EVs derived from neurons could regulate the differentiation of endogenous NSCs following SCI, considering that miRNA-21 has a key role in both the inflammatory process and nerve regeneration.

To answer these questions, we collected EVs from control or LPS-stimulated neurons and treated NSCs or SCI rats with these collected EVs. The results showed that EVs derived from control neurons have the ability to elicit NSC differentiation into oligodendrocytes and improve neurological functional recovery by promoting axonal regeneration and remyelinated around the lesion. In contrast, the EVs derived from LPS-stimulated neurons lost this capacity both in vitro and in vivo. In addition, we found that LPS stimulation of neurons could significantly upregulate miR-21 expression within EVs, which might be, at least in part, associated with the loss of their beneficial biological effects. Further study revealed that miR-21 could target SMAD 7 and upregulate the TGF-β/SMAD2 signaling pathway, inducing the differentiation of NSCs into astrocytes and preventing axonal remyelination.

## Methods

### Primary Culture Of Cortical Neuron, Neuron-Derived EVs Collection, And EVs Tracking

Primary cortical neurons were obtained from the cortex of newborn rat embryos, as described previously [35]. Briefly, cortical tissues were isolated and digested in trypsin for 30 min at 37 °C. The cell suspensions were incubated on culture flasks at a density of 1 × 10^6^/mL in DMEM/F12 culture medium (Gibco, USA) containing B27 (Gibco, USA). The culture medium was changed every three days.

The medium was changed to DMEM/F12 when 90% confluence was reached. After 24 h of culture, the supernatant was harvested as conditioned medium. To collect the neuron-derived EVs (neuron-EVs), the collected conditioned medium was centrifuged at 300 × g for 10 min, followed by further centrifugations at 2,000 × g for 20 min, and finally at 10,000 × g for 45 min at 4 °C to remove cell debris. Then, neuron-EVs were collected by centrifugation at 100,000 × g for 90 min at 4 °C. To identify neuron-EVs, Western blotting analysis was performed using the following primary antibodies: anti-CD63 (1:1000), anti-CD9 (1:1000), and anti- TSG101 (1:1000). Dynamic light scattering was used to detect the diameters of neuron-EVs (Supplementary Fig. 1). Dynamic light scattering showed that the particle concentration range was 2.2–2.8 × 10^9^ per 100 µl, and the BCA assay revealed that the protein concentration range within the EVs was 0.53–0.7 µg/µl. The collected neuron-EVs were dissolved in 100 µL of PBS and stored at − 80 °C. To collect the EVs derived from LPS-stimulated neurons (LPS-neuron-EVs), 10 ng/ml LPS (Sigma, Germany) was added to neuronal cultures at 90% confluence, and the neurons were cultured for 12 h. Then, the cells were washed three times with PBS and switched to DMEM/F12 for another 24 h of culture. The supernatant was obtained and concentrated to collect LPS-neuron-EVs.

For neuron-EVs tracking, the purified EVs were labeled with PKH-26 (Sigma, Germany) according to the manufacturer’s protocol. Briefly, 4 µl PKH-26 was diluted in 100 µl Diluent C and incubated with EVs for 10 min at room temperature. Next, these EVs were diluted in 1 ml PBS and collected by centrifugation at 100,000 × g for 90 min at 4ºC.

### NSC Culture And Transfection

NSC culture was performed as described in our previous studies [36, 37]. In brief, NSCs were harvested from the subventricular zone of SD rats and were suspended as neurospheres in DMEM/F12 containing 10 ng/mL basic fibroblast growth factor (bFGF, Gibco, USA), 20 ng/mL epidermal growth factor (EGF, Gibco, USA) and 2% B27 (Gibco, USA). 50nM miRNA-21-5p mimics (Guangzhou RuiBo, China) or 100 nM inhibitors (Guangzhou RuiBo, China) were used to upregulate or downregulate miR-21-5p expression in NSCs. A nontarget control miRNA mimic (mimic-NC, (Guangzhou RuiBo, China)) or a scrambled control sequence (inhibitor-NC, (Guangzhou RuiBo, China)) was performed as NCs or anti-NCs.

### Animal Experiments Protocols

Animal procedures were approved by the Ethics Committee of Anhui Medical University (No. 20,211,493) in accordance with the guidelines of the Declaration of Helsinki revised in Edinburgh in 2000. A weight-drop injury was performed to induce spinal cord injury at the T 9–10 level and a polyethylene catheter was inserted at the level of the injury for intrathecal injection (details are described in our previous studies [36, 37]). 25 µl of PBS, 25 µl of neuron-EVs, 25 µl of LPS-neuron-EVs, 50 nmol of miRNA-21-5p agomir (Guangzhou RuiBo, China), or 25 µl of LPS-neuron-EVs + 50 nmol of miRNA-21-5p antagomir (Guangzhou RuiBo, China) were administered to SCI rats via continuous injection. The inclined plane test and the Basso, Beattie, and Bresnahan (BBB) open-field test were performed to by two independent individuals evaluate the neurological outcome at different time points.

### Immunofluorescence Staining

The NSCs were dissociated into a single-cell suspension using trypsin and cultured on glass coverslips using medium containing 10% FBS-DMEM/F12. After 24 h of culture, the medium was switched to differentiation medium (DMEM/F12 containing 2% B27 and 1% antibiotic solution), or differentiation medium with neuron-EVs or differentiation medium with LPS-neuron-EVs. The medium was changed every three days. After seven days of culture, the cells were fixed for immunofluorescence staining. For tissue immunofluorescence staining, spinal cords were obtained from the SCI rats and incubated in 4% paraformaldehyde for 24 h. The spinal cords were then cut into a 4-µm-thick longitudinal slices (centered on the epicenter of the injured lesion) using a Leica RM2135 electric slicer (Leica, Germany), and these slices were prepared for immunofluorescence staining.

The details of immunofluorescence staining were described in our previous studies [36, 37]. The primary antibodies used for the staining were as follows: rabbit anti-glial fibrillary acidic protein (GFAP) for astroglia (1:1000; Abcam, United Kingdom), mouse anti-CNPase for oligodendrocytes (1:200; Abcam, United Kingdom), and rabbit anti-neuron-specific class III beta-tubulin (Tuj1) for neurons (1:1000; Abcam, United Kingdom). The secondary antibodies were Alexa Fluor 488 (green, 1:50; Elabscience, China) and Cy3 (red, 1:50; Elabscience, China). The stained slices were observed and photographed by using a DM-6B fluorescence microscope (Leica, Germany). For cell counting, random fields containing 300–500 cells were randomly selected. The percentage of positive cells in vitro experiments and the percentage positive areas in vivo experiments were calculated using ImageJ.

### Western Blot Assay

Cells, EVs or a 0.5 cm length section of injured spinal cord (centered on the epicenter of the injured lesion) were lysed in lysis buffer on ice. Ten micrograms of collected proteins were separated by sodium dodecyl sulfate–polyacrylamide gel electrophoresis (SDS‒PAGE) and transferred to a PVDF membrane. The membrane was then incubated with primary antibodies at 4 °C overnight (SMAD 7, 1:1000; p-Smad2, 1:1000, Invitrogen, USA) and with the secondary antibodies (Elabscience; 1:5000 in blocking solution) at room temperature for 1 h. The blots were then visualized using the SuperSignal West Pico enhanced chemoluminescence reagent (Advansta, USA) and quantified using ImageJ.

### RNA Extraction And Quantitative PCR

Cells from different groups and a 5-mm segment of spinal cord tissues (centered on the epicenter of the injured lesion) were collected for RNA extraction using TRIzol (Gibco, USA) according to the manufacturer’s instructions. cDNA was synthesized by using Superscript III RT Reaction Mix (Invitrogen). SYBR Green Master Mix (Applied Biosystems) and RealPlex2 Mastercycler (Eppendorf) were used to perform quantitative PCR. miRNA and mRNA expression were normalized to U6 and GAPDH, respectively. The sequences of transcript-specific primers were as follows: SMAD 7, 5′-GGGGGAACGAATTATCTGGC-3′, 5′- CGCCATCCACTTCCCTTGT-3′; GAPDH, 5′-CCGCATCTTCTTGTGCAGTG-3′, 5′- CGATACGGCCAAATCCGTTC-3′; miR-21-5p, 5′-TAGCTTATCAGACTGATGTTGA-3′; U6, 5′- CTCGCTTCGGCAGCACATATACT − 3′.

### Dual-Luciferase Reporter Analysis

The SMAD 7 3-UTR sequence containing the binding site of miRNA-21-5p to the target gene was cloned into a luciferase vector (GenScript, China) to create the SMAD 7-wt. In contrast, a mutant reporter of SMAD 7 was used to construct the SMAD 7-mut. NSCs were co-transfected with either the SMAD 7-wt or SMAD 7-mut, and miRNA-21-5p mimics or mimics-NC. After 48 h of transfection, luciferase activity was assessed using the Dual-Luciferase Reporter Assay System.

### Statistical Analysis

Statistical analysis was performed using SPSS software (version 16.0, USA). Data are presented as the mean ± standard deviation. Student’s t test (two groups) or one-way analysis of variance (ANOVA) (more than two groups) with Tukey’s post hoc method were used to evaluate the statistical significance. Statistical significance was set at a p values < 0.05.

## Results

### After LPS stimulation of neurons, the neuron-EVs lost their ability to promote NSC differentiation into oligodendrocytes

**Fig. 1:**
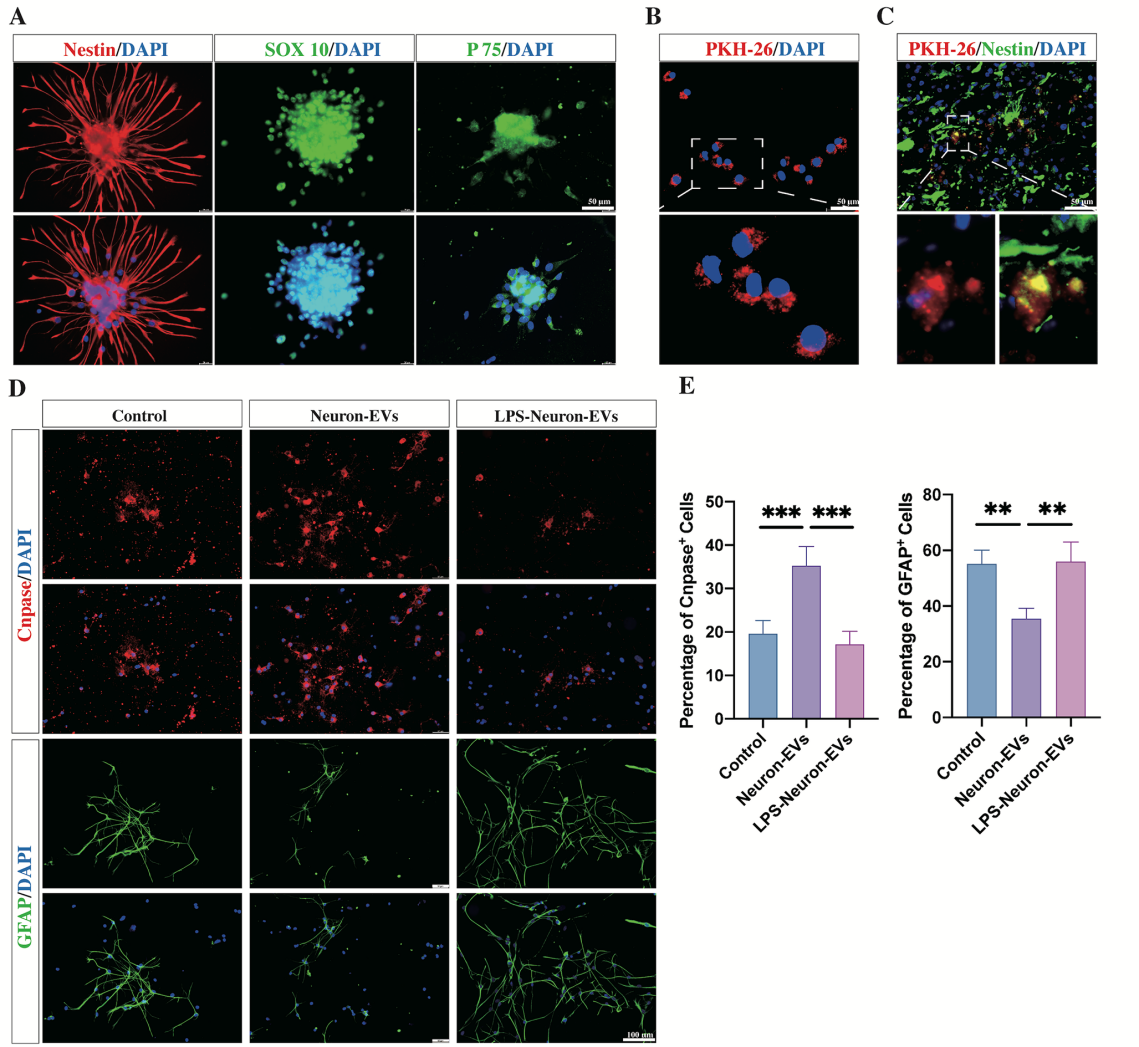
Effect of EVs released from non-stimulated or LPS-stimulated neurons on the differentiation of NSCs. (**A**) Immunostaining of NSCs with the markers Nestin, SOX 10, and P75 at Day 1. (**B**) PKH-26-labeled EVs were found within the cytoplasm of NSCs after 24 h of coculture (scale bars, 50 μm). The magnified views are displayed in the bottom panels. (**C**) Following the intrathecal injection of PKH-26-labeled EVs, PKH-26-labeled EVs (red) were found within Nestin-positive NSCs in the injured lesion sites at Day 3 postinjury (scale bar, 50 μm). The magnified views were displayed in the bottom panels. (**D**) After being cultured in the presence of neuron-derived EVs for seven days, the immunofluorescence results showed that the proportion of CNPase-positive oligodendrocytes was increased, and the proportion of GFAP-positive astrocytes was reduced compared to the control groups. In contrast, treatment with LPS-neuron-EVs did not modify the proportions of CNPase-positive oligodendrocytes and GFAP-positive astrocytes (scale bar, 100 μm). (**E**) Quantitation of CNPase-positive oligodendrocytes and GFAP-positive astrocytes in response to treatment of NSC cultures with either neuron-EVs or LPS-neuron-EVs (n = 5; data are the mean ± S.D.; ∗p < 0.05, **p < 0.001,***p < 0.0001)

To investigate whether LPS stimulation could affect the biological effects of neuron-EVs on the differentiation of NSCs, NSCs were cultured and identified by immunostaining with the markers Nestin, SOX 10, and P75 at Day 7 (Fig. 1A). Then, PKH-26 was used to label EVs derived from neurons. After 24 h of coculture with NSCs, the presence of labeled EVs (red) within the cytoplasm or surrounding the nucleus of NSCs was observed (Fig. 1.B). In vivo, three days after the SCI rats received an intrathecal injection of PKH-26-labeled neuron-EVs, immunostaining revealed that the Nestin-positive NSCs accumulated within the injured lesion site (Fig. 1.C). Moreover, PKH-26-labeled neuron-derived EVs (red) were found within the cytoplasm of these Nestin-positive cells (Fig. 1 C). These results indicated that EVs released from neurons could be taken up by NSCs both in vitro and in vivo.

Next, we cocultured NSCs with EVs derived from neurons for 7 days with differentiation medium. Immunostaining showed that the addition of neuron-EVs to NSCs resulted in an increase in the percentage of CNPase-positive oligodendrocytes and in a reduction in the percentage of GFAP-positive astrocytes (Fig. 1. D, E). Next, 10 ng/ml LPS was added to neuronal cultures. After 24 h, the EVs released from these cultures were collected and add to NSC cultures. Immunostaining showed that the addition of these EVs significantly reduced the proportion of oligodendrocytes from 35 to 17%, and increased the proportion of astrocytes from 35 to 55% (Fig. 1. D, E) compared to the previous condition.

**Fig. 2:**
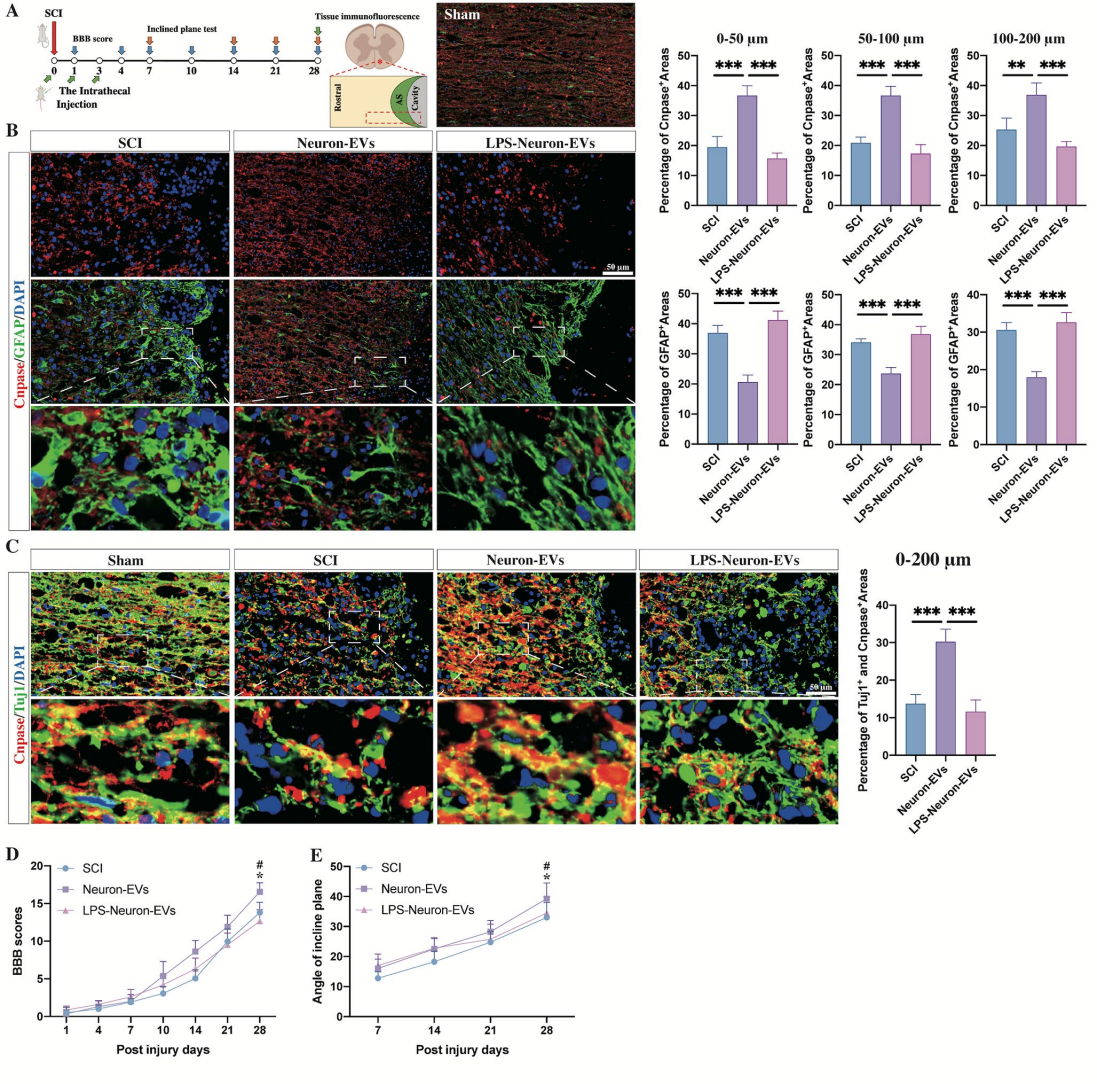
The effects of neuron-EVs and LPS-neuron-EVs on remyelination and neurological functional recovery following SCI. (**A**) Summary scheme of the experiment. View of the immunofluorescence stained section in the spinal cord. (**B**) Histograms showing the percentage of astrocytes (GFAP^+^) and oligodendrocytes (CNPase^+^) surrounding the cavity at 4 weeks postinjury in PBS-injected rats (SCI groups) and rats that received neuron-EVs, or LPS-neuron-EVs (n = 5, data are the mean ± S.D.; ∗p < 0.05, **p < 0.001,***p < 0.0001, ns p > 0.05; scale bar, 50 μm; bottom panels show magnified views; right panels show the quantitation of positive areas). (**C**) Immunostaining showing the percentage of remyelinated axons (Tuj1^+^ and CNPase^+^) around the cavity at 4 weeks postinjury in the rats that received injection of saline, neuron-EVs or LPS-neuron-EVs at 4 weeks postinjury (n = 5, data are the mean ± S.D.; ∗p < 0.05, **p < 0.001,***p < 0.0001, ns p > 0.05; scale bar, 50 μm; bottom panels, magnified views; right panels show the quantitation of positive areas). D, E. The BBB scores and incline plane tests at different time points following SCI (n = 10, data are the mean ± S. D, ∗ p < 0.05 control group vs. neuron-EVs, # p < 0.05 neuron-EVs vs. LPS-neuron-EVs).

Further in vivo immunostaining experiments showed that four weeks after the injury the center of the lesion site had formed a cavity surrounded by GFAP-positive astrocytic scars. Within these astrocytic boundaries, CNPase-positive oligodendrocytes were seldom observed (Fig. 2. B). In contrast, after a 3-day continuous intrathecal injection of neuron-EVs into SCI rats, significantly increased regrowth of oligodendrocytes through astrocytic scars was observed. However, the intrathecal injection of LPS-neuron-EVs into SCI rats failed to promote the regrowth of oligodendrocytes through the lesions. Instead, an astrocytic scar boundary was formed in the surrounding cavity (Fig. 2. B). In addition, remyelination of neurite outgrowths in or adjacent to the glial scars was determined via double-staining with Cnpase and Tuj1. The results revealed that the area that was positive for both CNPase and Tuj1 was significantly increased in the rats that received neuron-EVs treatment, compared to the untreated SCI rats. In contrast, the rats that received LPS-neuron-EVs treatment did not exhibit any increase in the double-positive area (Fig. 2. C). The neurological outcome was consistent with the histological data, with the rats that received injections of neuron-EVs exhibiting the highest scores for the BBB (Fig. 2. D) and angle inclined plane test (Fig. 2. E). In contrast, the injection of LPS-neuron-EVs did not promote neurological recovery (Fig. 2. D, E). Considered together, these pieces of data indicated that the neuron-EVs promote the differentiation of NSCs into oligodendrocytes and the regrowth of myelinated axons in SCI lesions, leading to an improvement in the neurological function. After LPS stimulation of neurons, however, the neuron-derived EVs lose the this capacity to promote NSC differentiation and remyelination following SCI.

### miR-21 inhibits the differentiation of NSCs into oligodendrocytes and axonal remyelination of axons within astrocytic scars

**Fig. 3:**
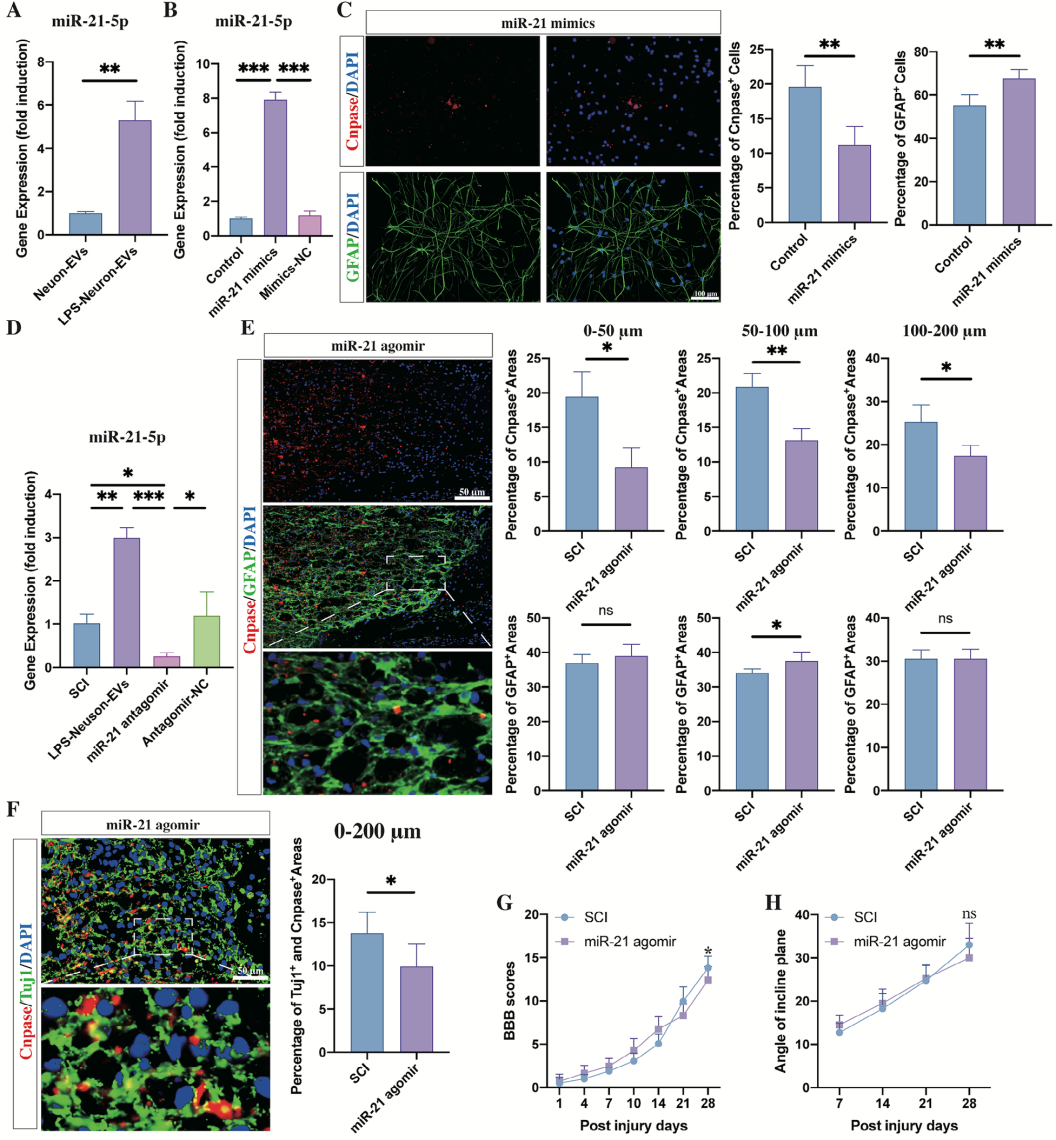
miR-21 promoted the differentiation of NSCs into astrocytes in vitro and induced remyelination failure following SCI. (**A**) The PCR analysis showed that the expression of miR-21 within neuron-EVs was upregulated by LPS stimulation (n = 3, data are the mean ± S. D, ∗p < 0.05, **p < 0.001,***p < 0.0001, ns p > 0.05). (**B**) The effect of the miR-21 mimics was confirmed by PCR after 24 h of transfection (n = 3, data are the mean ± S. D, ∗p < 0.05, **p < 0.001,***p < 0.0001, ns p > 0.05). (**C**) Transfection of miR-21 mimics promoted the differentiation of NSCs into astrocytes (n = 5; data are the mean ± S.D; *p < 0.05, **p < 0.001,***p < 0.0001, ns p > 0.05, scale bar, 100 μm; right panels show the quantitation of positive cells). (**D**) The effect of mir-21 agomir was confirmed by PCR after a 3-day continuous injection in SCI rats (n = 3, data are the mean ± S.D., ∗p < 0.05, **p < 0.001,***p < 0.0001, ns p > 0.05). (**E**) The expression of CNPase^+^ oligodendrocytes in the astrocytic scar boundary was inhibited by the injection of miR-21 agomir at week 4 post SCI (n = 5, data are the mean ± S.; ∗p < 0.05, **p < 0.001,***p < 0.0001, ns p > 0.05; scale bars, 50 μm; bottom panels show magnified views; right panels show the quantitation of positive areas). (**F**) The injection of miR-21 agomir significantly repressed the percentage of CNPase- and TUJ1-double-positive areas in lesion sites at week 4 post SCI (n = 5; data are the mean ± S.D.; *p < 0.05, **p < 0.001,***p < 0.0001, ns p > 0.05; scale bars, 50 μm; bottom panels show magnified views; right panels show the quantitation of positive areas). G, H. The BBB scores and incline plane tests at different time points following SCI in SCI rats and the rats that received miR-21 agomir injection (n = 10, data are the mean ± S. D, ∗ p < 0.05, ns p > 0.05)

Since miR-21 has been postulated to play a critical role in inflammation, a high miR-21 level in immune cells is considered to be a marker of the “active” state of these immune cells [32, 33]. Moreover, miR-21 has also been reported to be upregulated in the injured lesion sites following SCI [30]. Therefore, it is possible that inflammatory stimulation of neurons may upregulate the level of miR-21 within EVs and thus promote the differentiation of NSCs into astrocytes. To prove this possibility, we detected miR-21-5p expression in EVs derived from neurons stimulated with LPS. As expected, compared to EVs derived from non-stimulated neurons, miR-21-5p expression in these LPS-neuron-EVs was significantly increased (Fig. 3. A). Next, we tested whether miR-21-5p could mediate the differentiation of NSCs. We transfected NSCs with miR-21 mimics (Fig. 3. B) and calculated the percentage of astrocytes and oligodendrocytes after seven days of culture (Fig. 3. C). miR-21 transfection increased the proportion of astrocytes and decreased that of oligodendrocytes (Fig. 3. C). In vivo, we injected the miR-21 agomir to upregulate miR-21 expression (Fig. 3. D). Immunostaining revealed that the injection of miR-21 agomir markedly inhibited the regrowth of CNPase-positive oligodendrocytes (Fig. 3. E) and the remyelination of the neurite outgrowths (Fig. 3. F) within the astrocytic scar boundaries. The neurological assessment revealed that the injection of miR-21 worsened the outcome compared to the untreated SCI rats. This data indicates that LPS stimulation of neurons upregulate miR-21 expression within EVs, resulting in NSC differentiation into astrocytes and inhibition of oligodendrocyte regeneration following SCI.

### miR-21 Contributed To The loss Of The Biological Effects Of neuron-EVs

**Fig. 4:**
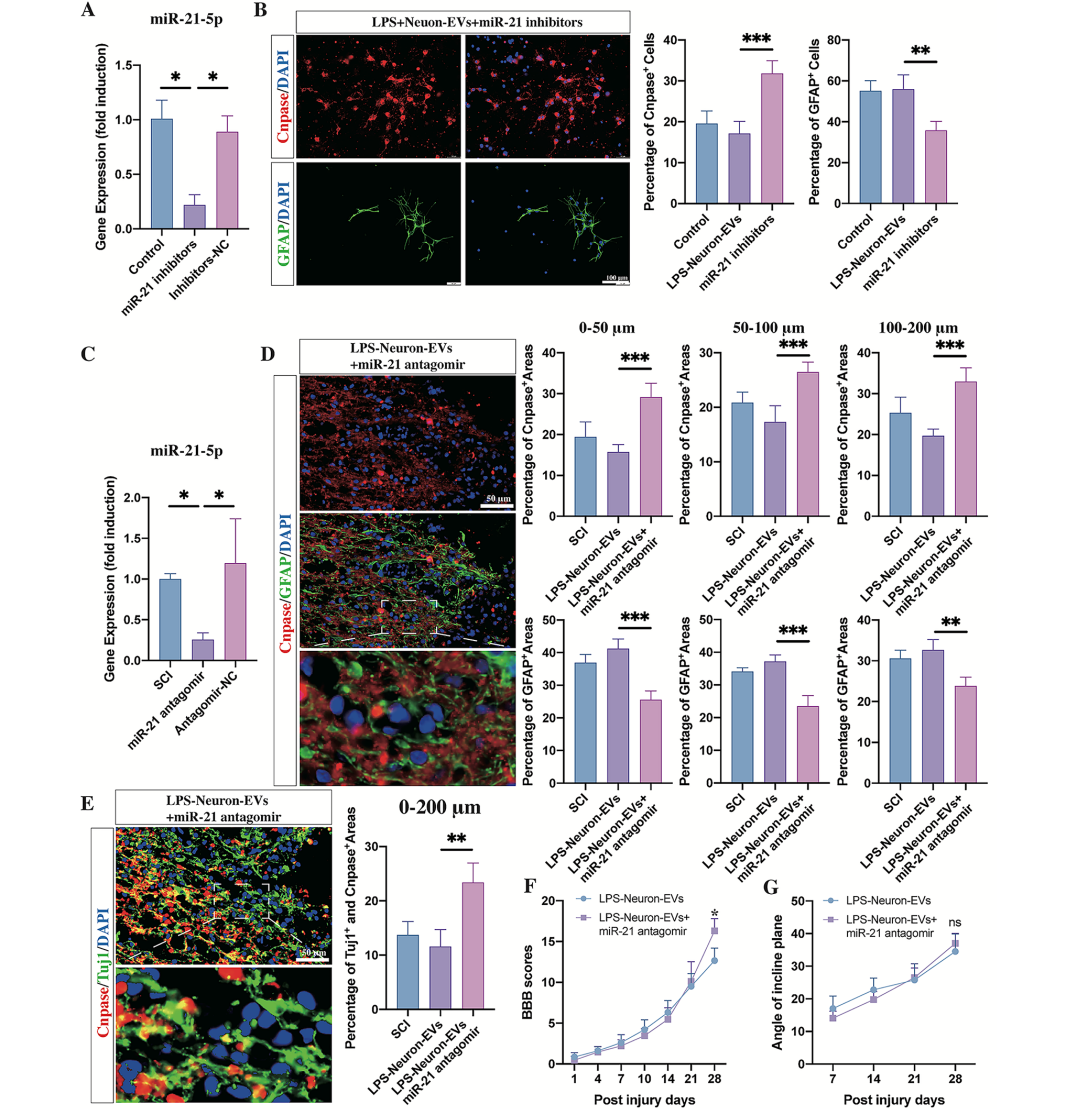
Upregulation of miR-21 Within Neuron-EVs Contributed to the Failure of Remyelination Following SCI (**A**) The effect of miR-21 antagomir was confirmed by PCR after 24 h of transfection (n = 3, data are the mean ± S. D, ∗p < 0.05, **p < 0.001, ***p < 0.0001, ns p > 0.05). (**B**) The transfection of miR-21 inhibitors markedly increased the percentage of CNPase^+^ cells in the presence of LPS-neuron-EVs (n = 5; data are the mean ± S.D.; *p < 0.05, **p < 0.001, ***p < 0.0001, ns p > 0.05, scale bar, 100 μm; right panels show the quantitation of positive cells). (**C**) The effect of mir-21 antagomir was confirmed by PCR after a 3-day continuous injection in SCI rats (n = 3, data are the mean ± S.D., ∗p < 0.05, **p < 0.001,***p < 0.0001, ns p > 0.05). (**D**) Injection of miR-21 antagomir inhibited the effects of LPS-neuron-EVs, leading to an increase in CNPase^+^ cells 4 weeks postinjury (n = 5; data are the mean ± S.D; *p < 0.05, **p < 0.001,***p < 0.0001, ns p > 0.05; scale bar, 50 μm; bottom panels show magnified views; right panels show the quantitation of positive areas). (**E**) The CNPase- and TUJ1-double-positive area was increased by the injection of miR-21 antagomir compared to the rats that received LPS-neuron-EV injection (n = 5; data are the mean ± S.D; *p < 0.05, **p < 0.001,***p < 0.0001, ns p > 0.05; scale bar, 50 μm; bottom panels show magnified views; right panels show the quantitation of positive areas). F, G. Compared to the rats that received LPS-neuron-EVS injection, the miR-21 antagomir- and LPS-neuron-EV-treated rats exhibited a better outcome in BBB scores and incline plane tests following SCI (n = 10, data are the mean ± S. D., ∗ p < 0.05, ns p > 0.05)

To further confirm whether the miR-21 enrichment was associated with the loss of the biological effects of neuron-EVs on NSC differentiation and neurological recovery in SCI rats, miR-21 inhibitors were transfected into NSCs (Fig. 4. A), and the cells were cultured for seven days in the presence of LPS-neuron-EVs. Immunostaining of the cultures showed that compared to NSCs cultured with LPS-neuron-EVs, the transfection of the miR-21 inhibitor increased the percentage of oligodendrocytes (Fig. 4. B). In vivo, the injection of miR-21 antagomir counteracted the effects of LPS-neuron-EVs on astrocytic scar formation, resulting in an increase in the axonal remyelination in the astrocytic boundary (Fig. 4. C, D, E). The neurological outcome was consistent with the histological data. The rats that received LPS-neuron-EVs and miR-21 antagomir injection demonstrated higher BBB scores (Fig. 4. F) and angle of incline plane (Fig. 4. G) compared to those that had only been treated with EVs derived from LPS-treated neurons.

### miR-21-5p Upregulates The TGF-β/Smad Signaling By Targeting SMAD 7

**Fig. 5:**
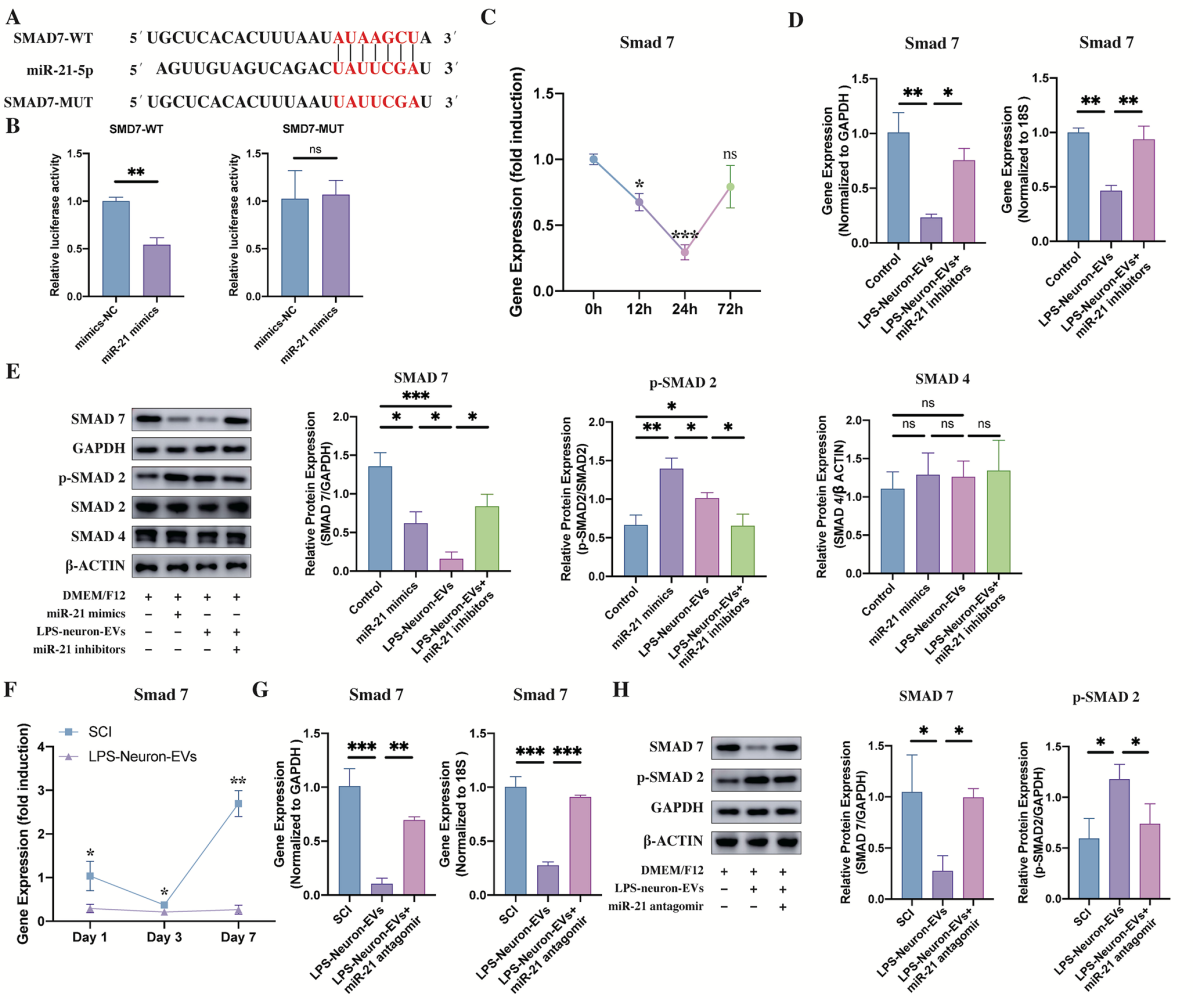
miR-21 regulates p-SMAD 2 expression by targeting SMAD 7 A. The target sequence for miR-21-5p in the 3′-UTR of SMAD 7 and the mutated target sequence. B. SMAD 7-wt or SAMD 7-mut were transfected into NSCs together with miR-21-5p mimics or mimics-NC. Dual luciferase reporter analysis confirmed the direct recognition of the SMAD 7 3′-UTR by miR-21-5p (n = 3; data are the mean ± S.D.; ∗p < 0.05, **p < 0.001, ***p < 0.0001, ns p > 0.05). C. PCR analysis showed that SMAD 7 expression in NSCs was downregulated by the addition of LPS-NSC-EVs (n = 3, data are the mean ± S. D, ∗p < 0.05, **p < 0.001, ***p < 0.0001, ns p > 0.05). D. The LPS-neuron-EV-induced downregulation of SMAD 7 in NSCs was abolished by the transfection of miR-21 inhibitors at 24 h of coculture (n = 3, data are the mean ± S. D, ∗p < 0.05, **p < 0.001, ***p < 0.0001, ns p > 0.05). E. Western blot analysis revealed that SMAD 7 expression was decreased and p-SMAD 2 expression was increased by the addition of LPS-neuron-EVs after 24 h of culture. Moreover, these LPS-neuron-EV-induced effects on the regulation of SMAD 7 and p-SAMD 2 expression were repressed by the addition of miR-21 inhibitors (n = 3, data are the mean ± S. D, ∗p < 0.05, **p < 0.001, ***p < 0.0001, ns p > 0.05). F. PCR confirmed that the expression of SMAD 7 was downregulated by the injection of LPS-neuron-EVs (n = 3, data are the mean ± S. D, ∗p < 0.05, **p < 0.001, ***p < 0.0001, ns p > 0.05). G. At Day 7 post SCI, the LPS-neuron-EV-induced downregulation of SMAD 7 was abolished by the injection of miR-21 antagomir (n = 3, data are the mean ± S. D, ∗p < 0.05, **p < 0.001, ***p < 0.0001, ns p > 0.05). H. Western blot analysis revealed that the injection of LPS-neuron-EVs reduced SMAD 7 expression while increasing p-SMAD 2 expression at Day 7 postinjury. In contrast, this LPS-neuron-EV-induced regulation of SMAD 7 and p-SMAD 2 could be abolished by the injection of miR-21 antagomir (n = 3, data are the mean ± S. D, ∗p < 0.05, **p < 0.001, ***p < 0.0001, ns p > 0.05)

To investigate the target gene of miR-21-5p, we used the TargetScan database to predict potential downstream gene candidates, this revealed that miR-21 was able to bind at positions 1182–1189 of the SMAD 7 3′UTR (Fig. 5. A). SMAD 7 works as a negative feedback regulator of transforming growth factor beta (TGF-β)/Smad signaling and plays a key role in astrocytic scar formation, axonal regrowth and remyelination. Therefore, we hypothesized that miR-21 may promote the differentiation of NSCs into astrocytes by inhibiting SMAD 7, thus preventing the SMAD7-induced negative regulation of TGF-β/Smad signaling.

To test this hypothesis, dual-luciferase reporter assays were used to characterize the interaction between miR-21-5p and SMAD7. Luciferase activity was decreased by the transfection of miR-21-5p mimics and SMAD7 3′UTR-wt (Fig. 5.B). In contrast, no difference in luciferase activity was noted in the NSCs transfected with miR-21-5p mimic- and SMAD7 3′UTR-mut (Fig. 5.B). This result indicated that SMAD 7 is the target gene of miR-21-5p. To test whether miR-21 could mediate the expression of SMAD 7, miR-21 mimics were transfected into NSCs, and the expression of SMAD 7 at different time points was evaluated by PCR. The expression of SMAD 7 was markedly reduced by the transfection of miR-21 mimics (Fig. 5C). Considering that SMAD 7 expression reached its lowest level at 24 h post transfection, we added LPS-neuron-EVs to NSCs and tested the expression of SMAD 7 and p-SMAD 2/3 (the downstream protein of TGF-β) after 24 h of culture. The expression of SMAD 7 was reduced, whereas that of p-SMAD 2 was increased by the addition of LPS-neuron-EVs (Fig. 5D, E). In contrast, the transfection of miR-21 inhibitors markedly counteracted the LPS-neuron-EVs-induced effects on the regulation of SMAD 7 and p-SMAD 2 expression, leading to an increase in SMAD 7 expression and a decrease in p-SMAD 2 expression (Fig. 5E). In addition, the expression of SMAD4 (which form a heterotrimeric complex with p-SMAD 2/3) remained unchanged between these groups (Fig. 5E).

We also detected the expression of SMAD 7 in the lesion sites at different time points following SCI. Compared to Day 1 postinjury, the expression of SMAD 7 was significantly increased at Day 7 postinjury (Fig. 5F). In contrast, the injection of LPS-neuron-EVs markedly inhibited SMAD 7 expression, especially at Day 7 postinjury (Fig. 5G). Then, we injected the miR-21 antagomir together with LPS-neurons-EVs and evaluated SMAD7 and p-SMAD 2 expression at Day 7 post injury. The expression of SMAD 7 was decreased, and the expression of p-SMAD 2 was increased following the injection of LPS-neuron-EVs (Fig. 5G, H). Moreover, this LPS-neuron-EVs-induced regulation was abolished by the co-injection of miR-21 antagomir. All these data suggested that the enrichment of miR-21 within LPS-neuron-EVs is responsible for the upregulation of TGF-β/Smad signaling, and that this is mediated by targeting SMAD 7 (Fig. 5G, H).

## Discussion

Posttraumatic inflammation plays a critical role in the secondary injury phase following SCI [38], triggering a series of pathophysiological processes and determining the final neurological functional outcome [39, 40]. The early appropriate inflammatory response can clear the cellular debris, which is considered to be beneficial to the regeneration of neural cells. Otherwise, without, or too little, inflammatory response might cause the accumulation of cellular debris in lesion sites, which are toxic to adjacent neural cells [41]. However, excessive inflammation might directly lead to damage to the sparing adjacent cells, resulting in the apoptosis of these cells [42, 43]. As a self-regulatory mechanism, inflammation induces endogenous NSCs to differentiate into astrocytes. This leads to the formation of astrocytic barriers surrounding the lesion core, limiting the spread of destructive inflammation to the neighboring tissue [10, 43, 44]. However, excessive formation of these astrocytic barriers also hinders the regeneration of axons toward the lesion sites, leading to the failure of neural circuit reorganization following SCI [10, 11]. In addition, the formation of astrocytic barriers requires a large number of endogenous NSCs, limiting oligodendrocyte differentiation due to the resulting shortage of precursors and therefore resulting in a remyelination impairment [7, 10]. Therefore, balancing astrocytic and oligodendrocytic differentiation is important for the reorganization of the neural circuits.

Emerging evidence has pointed out that cell‒cell communication through EVs participates in the regulation of pathological processes following neurotrauma. For example, in a proinflammatory scenario, EVs obtained from microglia inhibited remyelination at demyelinated lesions, while EVs isolated from microglia co-cultured with mesenchymal stem cells were able to promote myelin repair [45]. Similarly, studies have revealed that EVs produced by reactive astrocytes might inhibit neurite outgrowth and contribute to the neuronal apoptosis [46–49]. Lombardi et al. found that EVs produced by neurons were successfully transferred to astrocytes and mediate their biological activity [50]. In a model of peripheral nerve damage, Simeoli et al. revealed that EVs produced by capsaicin-stimulated neurons are taken up by nearby macrophages and promoted their polarization towards a proinflammatory phenotype [29]. In the present study, we found that EVs derived from neurons could directly influence the differentiation of NSCs. These EVs significantly promoted NSC differentiation toward oligodendrocytes in vitro and remyelination within astrocytic scars in vivo. However, the EVs produced by neurons subjected to proinflammatory conditions lost this capacity, resulting in a relatively larger astrocytic scar and in decreased remyelination surrounding the cavity. This result indicates that in response to inflammation, neurons send a signal to NSCs through EVs to promote NSC differentiation into more astrocytes, which induces the formation of glial barriers. This might be beneficial in the early phase of SCI, as it limits the spread of destructive inflammation. However, it also leads to the excessive formation of astrocytic barriers, resulting in axonal remyelination failure and worse neurological outcome.

In addition, several lines of evidence suggested that the loss of the neuron-EVs-induced effects on promoting NSC differentiation into oligodendrocytes might be associated with the upregulation of miR-21 within its EVs, caused by the stimulation of inflammation. First, the expression of miR-21 within neuron-EVs was significantly increased by LPS stimulation. Second, we showed that treatment with miR-21 mimics could increase the percentage of astrocytes and decrease the percentage of oligodendrocytes in vitro, as well as inhibit remyelination in the astrocytic scars surrounding the cavity following SCI, indicating that miR-21 acted as a remyelination inhibitor in the lesion sites. Moreover, further results revealed that the addition of miR-21 inhibitors or antagomirs abolished the LPS-neuron-EVs-induced astroglial differentiation of NSCs, partly rescuing the promoting effects of the EVs on remyelination.

MiRNAs are a class of small noncoding RNAs that are 20–22 nucleotides in length and enriched within EVs associated with cell‒cell communication. They negatively regulate the targeted mRNAs by degrading them or repressing their translation [14]. miR-21 has been shown to play a key role in inflammation. The overexpression of miR-21 in macrophages not only promotes their differentiation towards a proinflammatory phenotype but also increases the levels of proinflammatory cytokines they release [51]. A study by Su et al. revealed that miR-21 could mediate the phenotype of astrocytes following ischemic SCI. The upregulation of miR-21 in astrocytes causes their polarization from a proinflammatory neurotoxic reactive phenotype towards an anti-inflammatory neurotrophic reactive phenotype. Moreover, miR-21 silencing inhibited this polarization, resulting in the improvement of synaptic formation and nerve growth [34]. However, there is some evidence showing that miR-21 might have a dual role in the regulation the inflammatory response. Sheedy et al. revealed that miR-21 can directly target the proinflammatory protein PDCD4, which activates the transcription factor NF-κ. Thus, miR-21 inhibits the activation of this transcription factor and promotes the production of the anti-inflammatory cytokine IL-10, which in turn limits the inflammatory response induced by LPS [52]. In the present study, we found that miR-21 was upregulated in neuron-EVs by LPS stimulation. These miR-21-enriched EVs were taken up by NSCs, which were regulated to differentiate into astrocytes. Thus, miR-21 promoted the formation of astrocytic barriers, which contribute to the limitation of the spread of inflammation, indicating that miR-21 played an indirect anti-inflammatory role in the early phase of SCI. However, the upregulation of miR-21 in lesion sites was also associated with the excessive formation of astrocytic scars, leading to the failure of axonal remyelination and regrowth following SCI.

TGF-β plays an important role in the mediation of biological function in many systems, including the CNS [53, 54]. TGF-β can activate and phosphorylate the downstream protein Smad 2/3, which binds to the co-SMAD (SMAD 4) to form a complex that is then translocated to the nucleus to regulate gene expression [53, 54]. Previous studies have reported that the activation of the TGF-β signaling pathway promotes the formation of astrocytic scars following SCI [55, 56]. Our previous study found that the upregulation of TGF-β could directly mediate the differentiation of endogenous NSCs, promoting their differentiation to form astrocytic barriers following SCI [37]. In addition, TGF-β has been reported to increase the expression of chondroitin sulfate proteoglycans (CSPGs) in the lesion sites. CSPGs, known as axon inhibitors, accumulate around the lesion center and create an inhibitory environment for axonal regrowth [4, 57, 58]. In the present study, we found that miR-21 could target SMAD 7 to upregulate the TGF-β signaling pathway. SMAD 7 works as a negative regulator of TGF-β signaling by directly inhibiting the activation of its type I receptors. The activation of TGF-β could upregulate SMAD 7 expression, creating a feedback loop that prevents the over-activation of TGF-β signaling pathway [59, 60]. Therefore, the inhibition of SMAD 7 may lead to the amplification of TGF-β signaling. Herein, it was through the inhibition of SMAD 7 that miR-21 amplified the TGF-β signaling and thus promoted NSC differentiation into astrocytes.

In conclusion, following SCI, LPS stimulation of neurons upregulated miR-21 expression within neuron-EVs, which promoted NSC differentiation into astrocytes and the failure of axonal remyelination and regrowth targeting the SMAD7/TGF-β/SMAD2 axis.

## Electronic Supplementary Material

Below is the link to the electronic supplementary material.

Supplementary Material 1: Fig. 1 The identification of neuron-EVs. (**A**) Identification of BMSC-EVs by transmission electron microscopy. (**B**) Analysis of CD9, CD63, and TSG101 expression by western blot. (**C**) Detection of the diameter of BMSC- EVs by dynamic light scattering.

## Data Availability

The datasets used and/or analyzed during the current study are available from the corresponding author upon reasonable request.
