# Supplementary figures and images for: Inflammation Modifies miR-21 Expression Within Neuronal Extracellular Vesicles to Regulate Remyelination Following Spinal Cord Injury
Source: Stem Cell Rev Rep. 2023 May 31;19(6):2024–37. doi: 10.1007/s12015-023-10560-y (PMC10390616; doi:10.1007/s12015-023-10560-y)

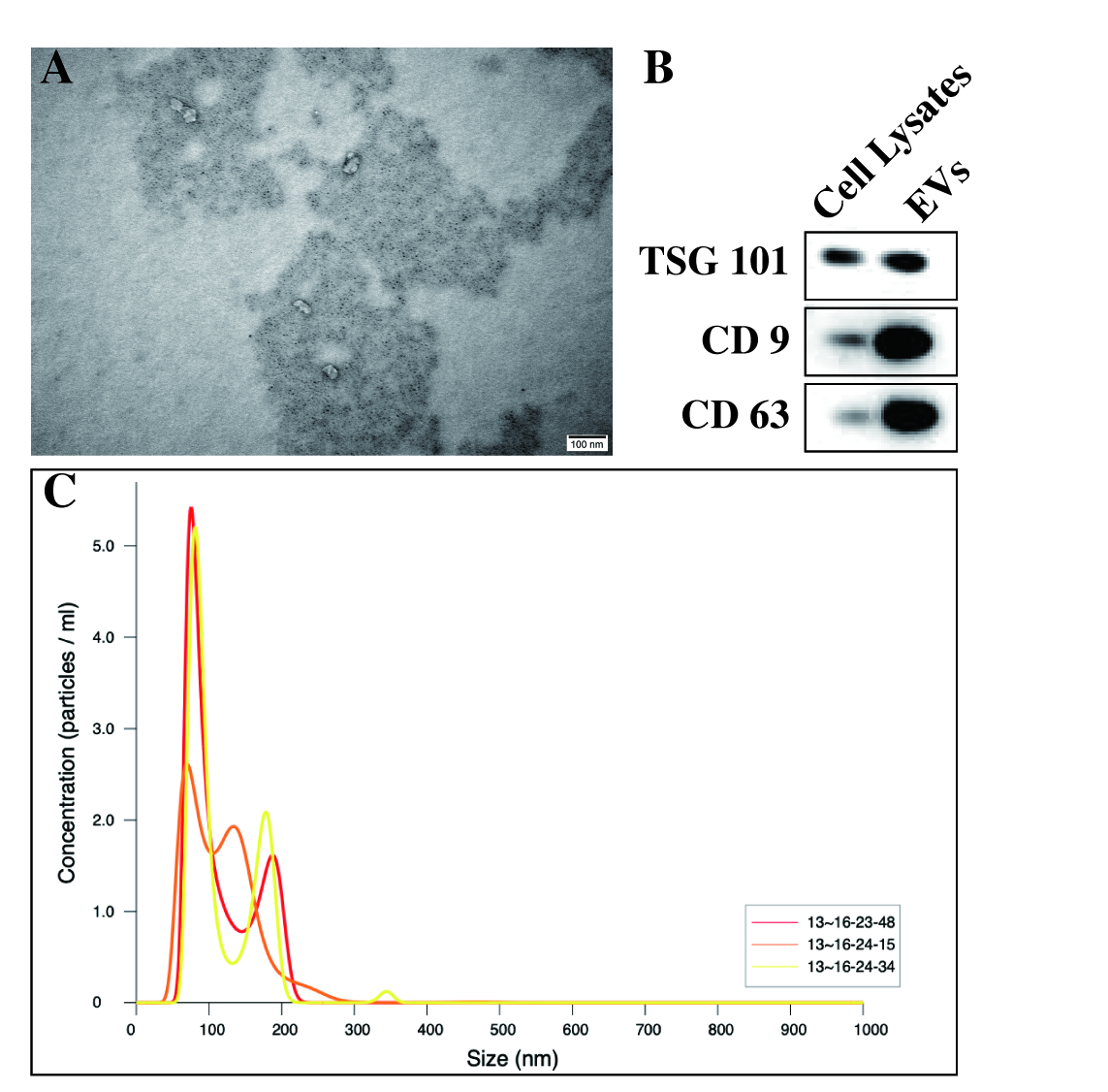

Supplement: Supplementary file 1 — Supplementary Material 1: Fig. 1 The identification of neuron-EVs. (A) Identification of BMSC-EVs by transmission electron microscopy. (B) Analysis of CD9, CD63, and TSG101 expression by western blot. (C) Detection of the diameter of BMSC- EVs by dynamic light scattering. [file 12015_2023_10560_MOESM1_ESM.tif]
